# A Putative *Plasmodium* RNA-Binding Protein Plays a Critical Role in Female Gamete Fertility and Parasite Transmission to the Mosquito Vector

**DOI:** 10.3389/fcell.2022.825247

**Published:** 2022-04-04

**Authors:** Sudhir Kumar, Biley A. Abatiyow, Meseret T. Haile, Kenza M. Z. Oualim, Amanda S. Leeb, Ashley M. Vaughan, Stefan H.I. Kappe

**Affiliations:** ^1^ Center for Global Infectious Disease Research, Seattle Children’s Research Institute, Seattle, WA, United States; ^2^ Department of Pediatrics, University of Washington, Seattle, WA, United States; ^3^ Department of Global Health, University of Washington, Seattle, WA, United States

**Keywords:** gametocyte, gamete, fertility, mosquito, transmission

## Abstract

*Plasmodium falciparum* sexual stage gametocytes are critical for parasite transmission from the human host to the mosquito vector. Mature gametocytes generate fertile male (micro-) or female (macro-) gametes upon activation inside the mosquito midgut. While a number of parasite genes have been described that are critical for *P. falciparum* gametogenesis and fertility, no parasite gene has been shown to have a unique function in macrogametes. The genome of *P. falciparum* encodes numerous RNA-binding proteins. We identified a novel protein containing a putative RNA-binding domain, which we named Macrogamete-Contributed Factor Essential for Transmission (MaCFET). This protein is expressed in the asexual and sexual stages. Parasites that carry a deletion of *MaCFET* (*Pfmacfet¯*), developed normally as asexual stages, indicating that its function is not essential for the asexual proliferation of the parasite *in vitro*. Furthermore, *Pfmacfet¯* male and female gametocytes developed normally and underwent activation to form microgametes and macrogametes. However, by utilizing genetic crosses, we demonstrate that *Pfmacfet¯* parasites suffer a complete female-specific defect in successful fertilization. Therefore, *Pf*MaCFET is a critical female-contributed factor for parasite transmission to the mosquito. Based on its putative RNA-binding properties, *Pf*MaCFET might be in involved in the regulation of mRNAs that encode female-specific functions for fertilization or female-contributed factors needed post fertilization.

## Introduction

*Plasmodium* is a digenetic parasite with a life cycle alternating between a vertebrate human host and a female *Anopheline* mosquito vector. While the majority of the erythrocytic stage parasites inside a human host grow asexually, developing through ring, trophozoite and schizont stages, some of the parasites differentiate into sexual stage gametocytes. *P. falciparum* gametocytes differentiate through stage I to stage V over a period of two weeks and show changes in their morphology and sexual commitment. The gametocyte sex ratio in *P. falciparum* is female biased, with both strain-specific and environmental factors playing a role in determining the actual ratio (Tadesse et al., 2019). When fully mature transmissible forms of stage V gametocytes are taken up by the mosquito vector in an infectious blood meal, they become activated to form gametes by a combination of factors including a drop in temperature (Sinden and Croll, 1975), an increase in pH (Sinden, 1983), and/or exposure to xanthurenic acid (XA), a metabolite of tryptophan (Billker et al., 1998). Gametogenesis is regulated by mobilization of intracellular calcium (Ca^2+^) stores, which control Ca^2+^ dependent protein function (Billker et al., 2004; Kumar et al., 2021) and is a rapid process with the male (micro) gametocyte undergoing three rounds of DNA replication and assembly of axonemes to form eight flagellar microgametes. The female (macro) gametocyte only undergoes a marked reduction in cytoplasmic density and nuclear changes to form a single macrogamete (Andreadaki et al., 2018). Microgametes display bidirectional flagellar motility through the blood meal, encounter macrogametes, and attach to initiate fertilization. Overall, fertilization represents a “bottleneck” in the parasite life cycle and is critical for transmission of the parasite (Kappe et al., 2010).

Cellular differentiation in organisms is controlled by “master” regulatory transcription factors and epigenetic mechanisms. While *Plasmodium* has a paucity of transcription factors, plant *Apetala*-related transcription factors have been identified to play a critical role for gametocytogenesis (López-Barragán et al., 2011; van Biljon et al., 2019). Since the transition from mature stage V gametocytes to fertilization-competent gametes is a rapid process, it can be assumed that the molecular framework to support this transition is already established in stage V gametocytes and is ready to be triggered by the aforementioned factors. However, little is known about the molecules that regulate this process.

RNA binding proteins (RBPs) regulate mRNA homeostasis and translation in various organisms (Nguyen-Chi and Morello, 2011; Oliveira et al., 2017; Díaz-Muñoz and Turner, 2018). In higher eukaryotes, RBPs control posttranscriptional gene regulation by binding to the 3′ UTR region of mRNAs and regulate germ cell decisions (Rangan et al., 2008; Kimble, 2011). In metazoans, transcripts required for germ cell specification and maternal to zygote transition are stored in ribonucleoprotein particles (mRNPs) called P granules (Stitzel and Seydoux, 2007; Boag et al., 2008; Voronina et al., 2011; Mukherjee and Mukherjee, 2021). In rodent malaria *Plasmodium* parasites, P granules and their components have been identified (Mair et al., 2010) and two of these components- the RNA helicase DOZI (Development Of Zygote Inhibited) and the Sm-like factor CITH (homolog of worm CAR-I and fly Tailer Hitch) control zygote to ookinete transition (Mair et al., 2010) by translationally repressing mRNAs contributed by the macrogamete (Mair et al., 2006; Mair et al., 2010).

The *Plasmodium* genome encodes 988 RBP candidates which correspond to 18.1% of the total *P. falciparum* proteome and 199 proteins of these interact with mRNA during the blood stages (Bunnik et al., 2016). Several of these RBP show elevated expression in gametocytes (Reddy et al., 2015; Bunnik et al., 2016). We have herein characterized one of these RNA-recognition motif (RRM) domain-containing proteins in *P. falciparum* and show that it is expressed in both asexual and sexual stages. Gene knockout parasites grow normally as asexual stages and develop and differentiate into mature gametocytes. These parasites also do not show any defect in male and female gametogenesis. However, they suffer a complete female-specific defect in successful fertilization. Therefore, we named this protein Macrogamete-Contributed Factor Essential for Transmission (MaCFET) as it is a critical female-contributed factor for transmission to the mosquito.

## Results

### *Pf*MaCFET is Expressed in Asexual and Sexual Stages

**FIGURE 1:**
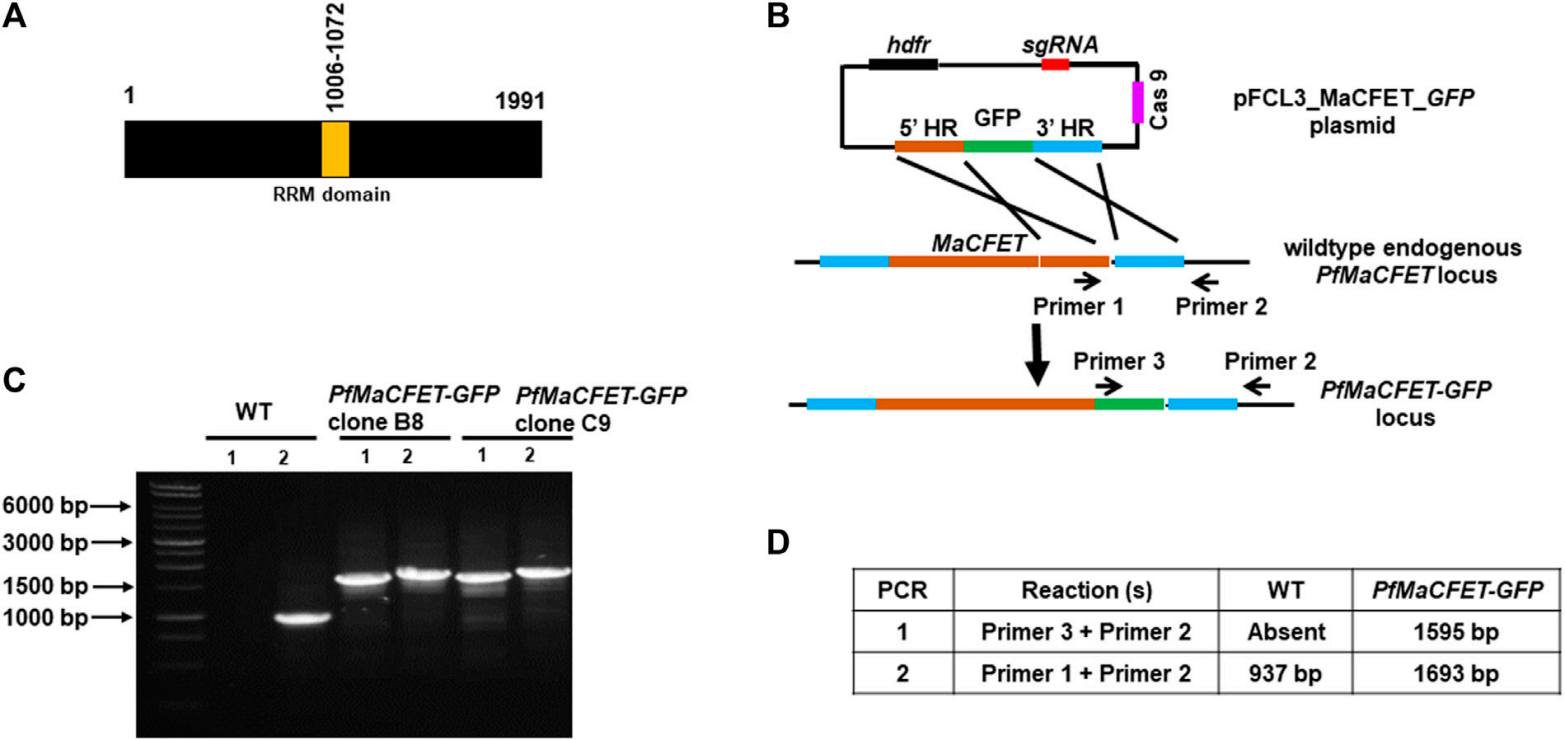
Generation of PfMaCFET-GFP parasites. **(A)** Schematic for *Pf*MaCFET domains showing an RNA recognition motif (RRM) domain. **(B)** Schematic for endogenous tagging of the *PfMaCFET* locus with GFP. The pFCL3_MaCFET_GFP plasmid contains homology arms from the 5′ (5′HR) and 3′ (3′HR) regions of the *PfMaCFET* locus, a single guide RNA seq (sgRNA), Cas9 and human dihydrofolate reductase (hDHFR). **(C)** Confirmation of *PfMaCFET-GFP* parasite generation by genotyping PCR. The oligonucleotides were designed and positions are indicated by arrows in **(B)** to confirm the introduction of the GFP tag. **(D)** The expected amplicon sizes for different sets of PCR primer combinations are indicated.

**FIGURE 2:**
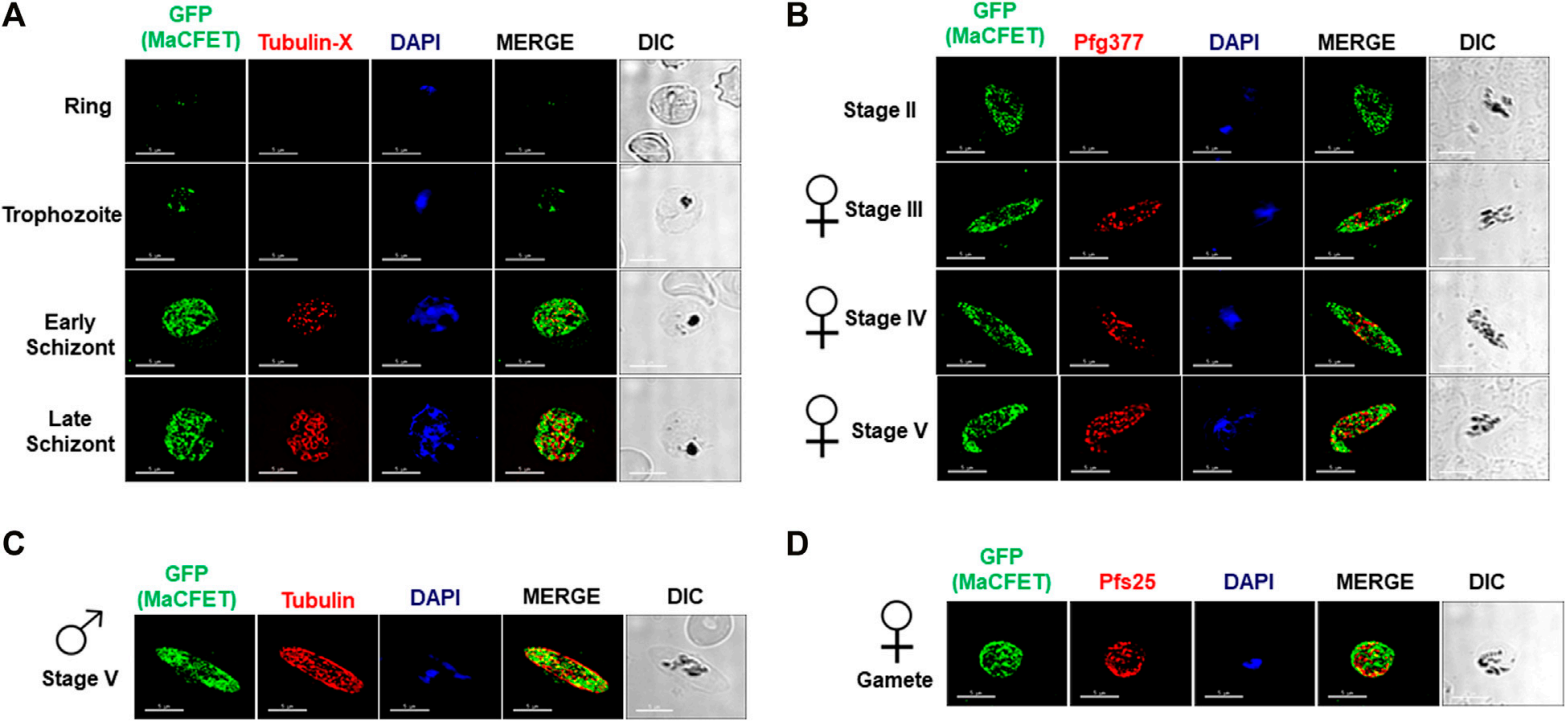
Expression and localization of PfMaCFET in asexual and sexual stage parasites **(A)** IFAs were performed using thin culture smears of asexual stages (ring, trophozoite, early and late schizonts), **(B)** sexual (stage II-V gametocytes) stages, **(C)** male gametocyte, **(D)** female gamete using anti-GFP antibody (in green) in combination with anti-Tubulin X **(A)**, anti-PfG377 **(B)**, anti-Tubulin **(C)** anti-Pfs25 **(D)**. The parasite DNA was visualized with DAPI (blue). Representative images are shown. DIC, differential interference contrast. DAPI, 4′,6-diamidino-2-phenylindole, Scale bar = 5 μm.

Recent transcriptomic studies on gametocyte stages have identified several genes exhibiting high expression (López-Barragán et al., 2011; Lasonder et al., 2016; van Biljon et al., 2019). Among these genes, Pf3D7_1241400 showed high expression in gametocyte stages compared to asexual stages (van Biljon et al., 2019). Since the gene deletion parasites (described below) for this gene had a complete block in macrogamete fertility, we named it Macrogamete-Contributed Factor Essential for Transmission (MaCFET). Domain analysis for this protein revealed the presence of a single RRM domain (Figure 1A). To analyze *Pf*MaCFET expression, we generated transgenic parasites in the *Pf*NF54 strain with a GFP tag at the C-terminus of the protein (Figures 1B–D). Indirect immunofluorescence assays (IFAs) were performed on thin parasite culture smears and stained with anti-GFP antibodies to detect *Pf*MaCFET and anti-Tubulin-X to mark parasite microtubules demonstrating that *Pf*MaCFET expression throughout intraerythrocytic parasite development (Figure 2A). *Pf*MaCFET localized within the parasite cytoplasm and the distribution of the protein had a granular appearance. *Pf*MaCFET was also detected in stage II through stage V gametocytes (Figure 2B). Co-staining with macrogametocyte-specific antibodies (anti-Pfg377) (Figure 2B) or microgametocyte -specific antibodies (anti-Tubulin) (Figure 2C), or macrogamete antibodies (anti-Pfs25) (Figure 2D) revealed that *Pf*MaCFET is expressed in both male and female gametocytes. These results show that *Pf*MaCFET is expressed in both asexual and sexual stages of the parasite.

### *Pfmacfet¯* Gametocytes Do Not Display Any Defect in Asexual Stage Replication and Sexual Stage Formation

**FIGURE 3:**
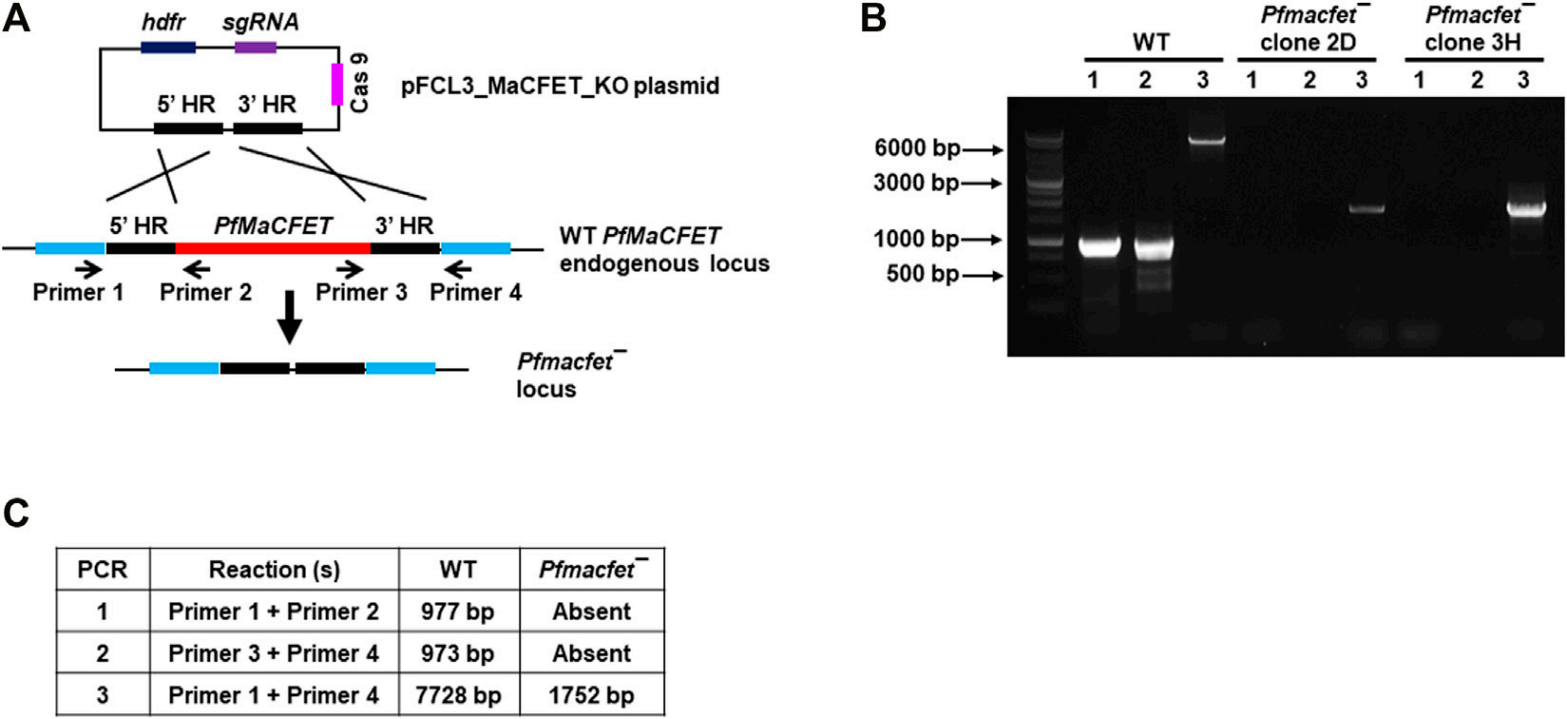
Generation of *Pfmacfet¯* parasites. **(A)** For generation of *Pfmacfet¯* parasites a similar strategy as described in Figure 1 was used. pFCL3_PfMaCFET_KO plasmids had homology regions from 5′ (5′HR) and 3′ (3′HR) of the *PfMaCFET* locus, a single guide RNA seq (sgRNA) and human dihydrofolate reductase (hDHFR). **(B,C)** *PfMaCFET* deletion confirmation *via* diagnostic PCR. The oligonucleotides were designed from outside the 5′HR and 3′HR and the *PfMaCFET* locus and positions are indicated by arrows in **(A)**. The expected amplicon sizes for different set of PCR primer combinations are indicated in **(B,C)**.

For functional analysis, the endogenous *PfMaCFET* gene was deleted using CRISPR/Cas9 methodology. Genomic regions (800–1,000 bp) from upstream (5′) and downstream (3′) of the *PfMaCFET* genomic region were PCR-amplified, ligated through an overlapping linker region and then cloned in pFCEF1α-Linker3 vector (Figure 3A). The plasmid was transfected using BIO-RAD gene pulsar using standard methods. Parasites were selected using WR99210 (8 µM). Parasites which appeared after drug selection were used to harvest genomic DNA. Deletion of the *PfMaCFET* locus was ascertained by a set of diagnostic PCRs with oligonucleotides specific for the *PfMaCFET* locus and its 5′ (upstream) and 3′ (downstream) genomic regions (Figures 3A–C). *Pfmacfet¯* parasites were cloned by limiting dilution and two clones were utilized for phenotypic analysis. Whole genome sequencing was performed on WT *Pf*NF54 and *Pfmacfet¯* parasites, which confirmed deletion of the *PfMaCFET* locus and showed no off-target genetic changes in other regions of the genome (data not shown).

**FIGURE 4:**
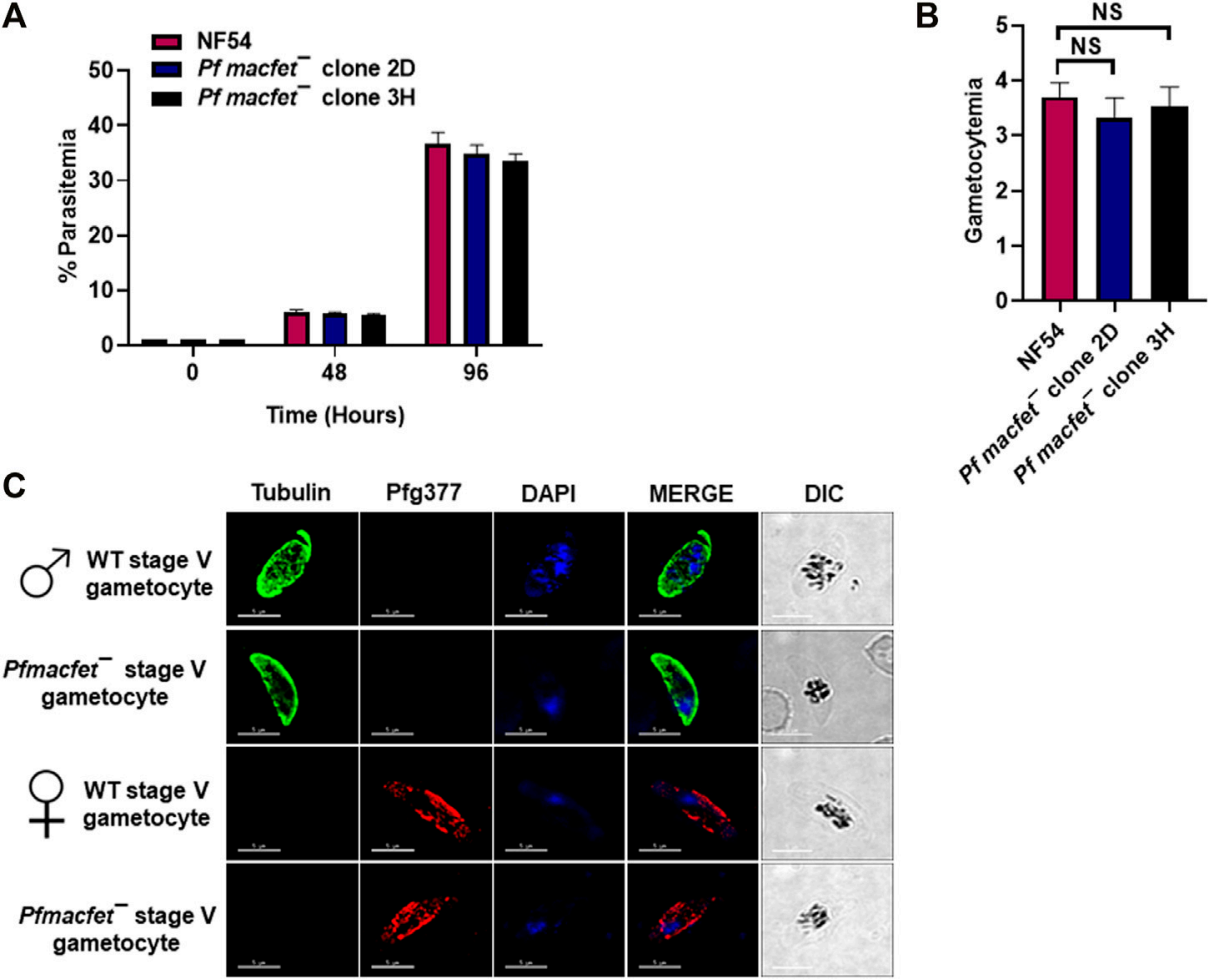
*Pfmacfet¯* parasites grow normally as asexual parasites and undergo gametocytogenesis. **(A)** *Pfmacfet¯* (clone 2D and 3H) parasites grew at a similar rate as WT *Pf*NF54. Data were averaged from three biological replicates and presented as the mean ± standard deviation (SD). **(B)** Bar graph shows gametocytemia for WT *Pf*NF54 and *Pfmacfet¯* parasites (clone 2D and 3H) measured on day 15. Data were averaged from three biological replicates and presented as the mean ± standard deviation (SD). **(C)** IFAs performed on thin blood culture smears for WT *Pf*NF54 or *Pfmacfet¯* (clone 3H) using α-tubulin (green), a male-specific marker, and *Pf*g377 (green), a marker for female gametocytes. Representative images are shown. DIC, differential interference contrast. DAPI, 4′,6-diamidino-2-phenylindole, Scale bar = 5 μm.

To detect potential defects the *PfMaCFET* deletion might cause in asexual blood stage growth, a comparative growth assay was set up for wildtype (WT) *Pf*NF54 and *Pfmacfet¯* parasites (clone 2D and 3H). Growth was monitored for two asexual cycles by enumerating parasitemia *via* Giemsa-stained culture smears. These experiments showed that *Pfmacfet¯* parasites do not suffer any apparent growth defect during asexual blood stage development (Figure 4A). We next evaluated the ability of *Pf macfet¯* parasites to undergo gametocytogenesis. For this, gametocytemia was induced for WT *Pf*NF54 and *Pf macfet¯* (clone 2D and 3H) as described before (Tripathi et al., 2020). The *Pfmacfet¯* parasites underwent gametocytogenesis and were able to develop into mature stage V male and female gametocytes (Figures 4B,C).

### *Pfmacfet¯* Gametocytes Undergo Gametogenesis

**FIGURE 5:**
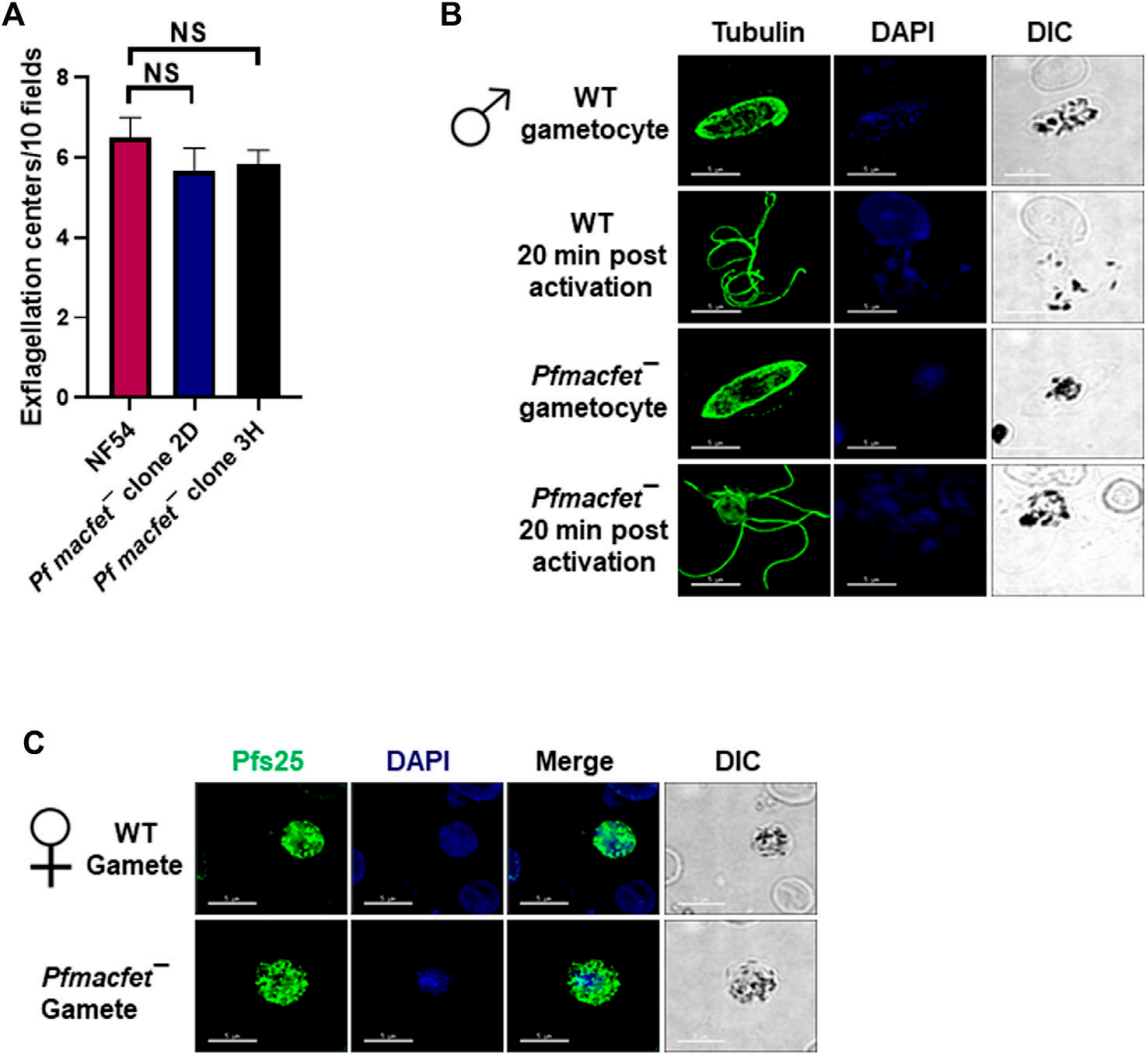
*Pfmacfet¯* parasites form male and female gametes. **(A)** Number of exflagellation centers (vigorous flagellar beating of microgametes in clusters of RBCs) per microscopic field at 15 min post-activation. Data were averaged from three biological replicates and presented as the mean ± standard deviation (SD). **(B)** IFAs performed on thin blood culture smears for gametocytes activated for 20 min *in vitro* for WT *Pf*NF54 or *Pfmacfet¯* (clone 3H) using α-tubulin II (green), a male-specific marker. α-Tubulin II staining showed microgametes emerging from exflagellating male gametocyte in both WT *Pf*NF54 and *Pfmacfet¯* gametocytes. **(C)** IFAs performed on thin blood culture smears for day 15 stage V gametocytes for WT *Pf*NF54 or *Pfmacfet¯* (clone 3H) using α-*Pf*s25 (green), a marker for female gametes. Representative images are shown. DIC, differential interference contrast. DAPI, 4′,6-diamidino-2-phenylindole, Scale bar = 5 μm.

We next analyzed the ability of *Pfmacfet¯* parasites to undergo gametogenesis. Day 15 gametocyte cultures for WT *Pf*NF54 and *Pfmacfet¯* (3H) were activated by adding O^+^ human serum and a decrease in temperature from 37°C to room temperature (RT). To investigate male gametogenesis, the activated gametocytes were mounted on glass slides and exflagellation centers were measured in 10 random fields of view using brightfield microscopic illumination at ×40 magnification. A similar number of exflagellation centers were observed for WT *Pf*NF54 and *Pfmacfet¯* parasites, indicating normal male gametogenesis (Figure 5A). We further performed IFAs using anti-α-Tubulin antibodies to stain male gametocytes and male gametes and reveal the formation of flagellated microgametes. This showed that *Pfmacfet¯* microgametes (exflagellae) formed normally and egressed the gametocyte body and did not show any apparent morphological defect when compared to WT *Pf*NF54 microgametes (Figure 5B). We then performed IFAs using anti-*Pf*s25 antibodies to stain macrogametes, which revealed that *Pfmacfet¯* parasites were able to form macrogametes similar to WT *Pf*NF54 parasites (Figure 5C). These results indicated *Pf*MaCFET is not important for male or female gametogenesis.

### *Pfmacfet¯* Parasites Cannot Infect the Mosquito Vector

**FIGURE 6:**
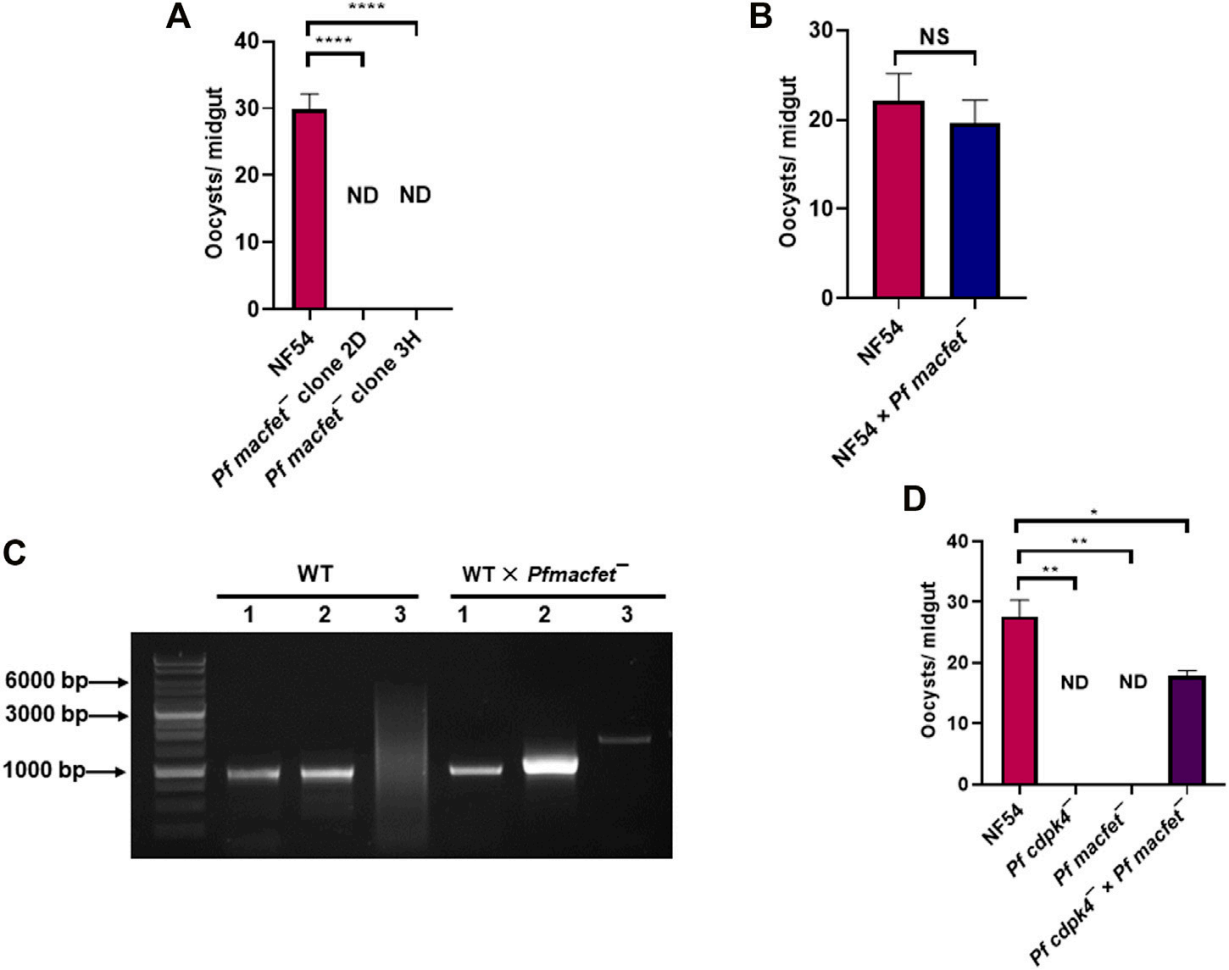
*Pf*MaCFET regulates fertility of female gametes. **(A)** Mosquitoes with single parasite lines or genetic crosses were dissected on day 7 post feed and number of oocysts were measured per midgut. Data were averaged from three biological replicates with a minimum of 50 mosquito guts and presented as the mean ± standard deviation (SD) *n* = 3. ****p-value= <0.0001. **(B)** Oocyst numbers for WT *Pf*NF54 and the WT *Pf* NF54 × *Pfmacfet¯* cross. Error bar indicates mean ± SD; *n* = 2. NS-Not significant. **(C)** Confirmation of transmission of *Pfmacfet¯* parasites in a WT × *Pfmacfet¯* clone 3H genetic cross by genotyping PCR. The oligonucleotides were designed from outside the 5′ and 3′ homology regions of the *PfMaCFET* locus and positions as indicated by arrows in Figure 3A. **(D)** Oocyst formation for WT *Pf*NF54, *Pfmacfet¯*, *Pfcdpk4¯* single parasite infections and the *Pfmacfet¯* × *Pfcdpk4¯* genetic cross infection. Error bar indicates mean ± SD; *n* = 2. ND-Not detected, **p-value = 0.0048, *p-value = 0.0399.

We next examined the transmissibility of *Pfmacfet¯* gametocytes to *Anopheles stephensi* mosquitoes. Infectious blood meals were prepared by mixing WT *Pf*NF54 and *Pfmacfet¯* stage V gametocytes with human serum and RBCs which were then loaded into standard membrane feeders. WT *Pf*NF54 parasite feeds resulted in an average oocyst number of ∼30/mosquito, while *Pfmacfet¯* parasite feeds did not show any oocysts in mosquito midguts for both *Pfmacfet¯* clones (Figure 6A). This complete block in transmissibility of *Pfmacfet¯* parasites could be due to a defect in either microgamete or macrogamete fertility or defects in both genders. To analyze gender-specific defects in fertility, a genetic cross was first performed between WT *Pf*NF54 and *Pfmacfet¯* gametocytes by mixing the mature stage V gametocyte cultures and feeding them to the same mosquitoes. Mosquitoes were dissected day 7 post feed to obtain infected midguts which revealed similar number of developing oocysts for WT *Pf*NF54 × *Pfmacfet¯* and WT *Pf*NF54 only control feeds (Figure 6B). These infected mosquito midguts with developing oocysts stages were then used to isolate genomic DNA. Genotyping PCR using primer pairs shown in Figure 3A demonstrated the presence of *Pfmacfet¯* parasites in WT *Pf*NF54 × *Pfmacfet¯* fed mosquito midguts (Figure 6C). These results indicate that one of the genders of *Pfmacfet¯* must be fertile. Genotyping PCRs performed on sporozoite stages confirmed these results, showing that *Pfmacfet¯* parasite detection was not due to residual parasites in the blood meal (data not shown).

To ascertain the gender-specific function of *Pf*MaCFET, we assessed the fertility of microgametes and macrogametes of *Pfmacfet¯* using an additional genetic cross. The cross was arranged between *Pfmacfet¯* parasites and a gene deletion parasite line of the *CDPK4* locus, which we have shown generates only fertile macrogametes (*Pfcdpk4¯*) (Kumar et al., 2021). WT *Pf*NF54, *Pfcdpk4¯* and *Pfmacfet¯* gametocytes were generated *in vitro* and first fed individually to female *A. stephensi* mosquitoes on day 15 of culture. Mosquitoes were dissected seven days post feed to enumerate oocysts in the midgut. As expected, WT PfNF54 gametocytes showed robust mosquito midgut infection but *Pfcdpk4*¯ and *Pfmacfet*¯ fed mosquitoes showed no oocysts in the midgut (Figure 6D). Next the *Pfcdpk4¯* × *Pfmacfet¯* cross was evaluated and showed robust mosquito midgut oocyst infection, albeit at reduced levels compared to WT *Pf*NF54. Together, these results demonstrated that *Pfmacfet¯* parasites suffered a macrogamete-specific defect in productive fertilization.

## Discussion

*Plasmodium* transmission to the mosquito vector requires differentiation of sexually replicating parasites into gametocytes. Only at stage V, are gametocytes distinguishable as male or female genders. Further differentiation of gametocytes into gametes requires sex-specific gene expression (Guttery et al., 2015). However, how these parasites express distinct transcripts in the male and female gametocytes remain largely unknown. In the rodent malaria model parasite *P. yoelii*, the AP2 family protein AP2-FG and AP2-O3 have been shown to play a role in differentiation of early gametocytes to the female lineage by governing a female-specific gene expression repertoire (Yuda et al., 2020; Li et al., 2021). Work mostly done in another rodent malaria parasite *P. berghei* showed that gametocytes also employ translational repression (Mair et al., 2006) and inactive mRNPs (P granules) store several macrogamete specific transcripts which are stabilized by DOZI and CITH (Mair et al., 2010). Several RNA binding proteins are also part of this complex (Mair et al., 2010). Although there are numerous RNA binding proteins encoded in the *Plasmodium* genome (Reddy et al., 2015), none of them have been identified to play role in gamete fertility. Here, we show that the putative RNA binding protein *Pf*MaCFET is essential for fertility of macrogametes and deletion of the coding gene blocks parasite transmission to the mosquito in a female-gender-specific manner.

*Pf*MaCFET expression is highest in gametocytes (PlasmoDB). In this study, we show that *Pf*MaCFET is expressed in ring, trophozoites and schizont stages of asexual parasite development as well as in stage II- stage V gametocytes. *Pf*MaCFET primarily displayed a granular cytoplasmic localization in asexual and sexual stages. Expression in sexual-stage parasites was not sex-specific as both mature male gametocytes and female gametocytes and gametes expressed *Pf*MaCFET.

Gene deletion of *PfMaCFET*, revealed that although *Pf*MaCFET is expressed in asexual blood stages and throughout gametocyte development, it is not required for asexual blood stage replication or complete gametocyte development. We found that *Pfmacfet¯* parasites developed into mature stage V male and female gametocytes and underwent gametogenesis. We also observed no discernible defect in *Pfmacfet¯* microgamete and macrogamete formation. *Pf macfet¯* parasites did not infect the mosquito vector but strikingly, when crossed with WT *Pf*NF54 gametocytes, infected mosquitoes were shown to carry *Pfmacfet¯* parasites. This demonstrated that one gender of *Pfmacfet¯* gametes must be fertile. An additional genetic crosse, utilizing a male sterile line (*Pfcdpk4¯*) (Kumar et al., 2021) and *Pfmacfet¯*, revealed that *Pfmacfet¯* male gametes are fertile but female gametes are sterile.

Previous studies involving sex-specific proteomes have identified 1,244 and 1,387 proteins in mature *Pf* male and female gametocytes, respectively (Miao et al., 2017). These studies revealed that the male-specific proteome is enriched in proteins associated with flagellum formation and genome replication while the female-specific proteome is more abundant in proteins involved in metabolism and translation (Miao et al., 2017). *Pf*MaCFET contains an RRM domain which suggests an RNA-binding function. It is thus reasonable to suggest that *Pfmacfet¯* macrogametes are sterile due to perturbations of the macrogamete transcriptome. This however requires further analysis. *Pf*DOZI and *Pf*CITH show increased expression in macrogametocytes (PlasmoDB) and in rodent malaria parasites are part of a complex that stabilizes and translationally represses numerous transcripts which are maternally derived and critical to zygote formation (Mair et al., 2010). *Pf* eukaryotic initiation factor 4E (*Pf*eIF4E) interacts with *PfDOZI* in asexual blood stages and regulates its translation inhibitory activity (Tarique et al., 2013). It is possible that this association of *Pf*eIF4E and *PfDOZI* continues in sexual gametocyte stages. *Pf*MaCFET might be part of these complexes where it might bind and stabilize transcripts *via* its RRM domain and thereby regulate the fertility of macrogametes. *Pf*MaCFET is, to our knowledge, the first female-specific fertility determining factor in *P. falciparum. Pfmacfet¯* parasites will be a powerful tool to study the gender-fertility related functions of *P. falciparum* genes via genetic crosses. Further studies are warranted to identify the molecular mechanisms of *Pf*MaCFET function and the pathways by which it regulates female fertility.

## Methods

### Reagents and Primary Antibodies

All molecular biology reagents and oligonucleotides were purchased from MilliporeSigma, US until otherwise stated. The following primary antibodies, antisera and dilutions were utilized: rabbit α-Pfg377 (1:500, kindly gifted by Professor Pietro Alano at Istituto Superiore di Sanità, Italy), mouse α-tubulin antibody (1:200, Millipore SIGMA, cat# T5168), rat tubulin-X antibody (1:100, Millipore SIGMA, cat# MAB 1864).

### *Plasmodium falciparum* Culture and Transfection

Standard procedures were followed to culture *Pf* parasites (WT *Pf*NF54 and *Pfmacfet¯*) as asexual blood stages and were given complete RPMI media, supplemented with either 0.5% AlbuMAX^TM^ II (Thermo Scientific) medium or 10% (v/v) human serum every 24 h. *In vitro* gametocytes were generated using O+ human RBCs (Valley Biomedical, VA, US) and O+ human serum (Interstate Blood Bank, TN, US) using methods published elsewhere (Tripathi et al., 2020).

Oligonucleotides used for the creation and analysis of *Pf*MaCFET-GFP parasites are detailed in Table 1. Successful tagging at 3′ of MaCFET was confirmed by a set of genotyping PCRs (Figure 1B). Oligonucleotides used for the creation and analysis of *Pfmacfet¯* parasites are detailed in Table 1. Deletion of *PfMaCFET* (PlasmoDB identifier Gene—PF3D7_1241400) was achieved using standard methods. Gene deletion was shown by a set of genotyping PCRs (Figure 3B). Whole genome sequencing was performed on WT *Pf*NF54 and *Pfmacfet¯* parasites, which confirmed deletion of the *PfMaCFET* locus and showed no off-target genetic changes in other regions of the genome (data not shown). Two individual clones for *Pfmacfet¯* (clone 2D and 3H) were chosen for phenotypic characterization.

**TABLE 1:** Oligonucleotides used in the study.

Oligonucleotides used for generation of *Pf macfet¯* parasites	
Oligo	Forward (5′-3′)
PfMaCFET 5′Homo For	TGC​GGC​CGC​GTT​GAC​TAA​ATA​ATT​TTG​AGG​TAT​TCC​ATT​AG
PfMaCFET 5′Homo Rev	CCA​ACC​CGG​GTA​TAG​GCG​CGC​CTG​GAA​GAG​GAA​AAA​TAA​ATG​TAT​ATA​CGG​C
PfMaCFET 3′Homo For	AGG​CGC​GCC​TAT​ACC​CGG​GTT​GGT​ATG​TAT​ATT​ATA​GCA​CAT​GGT​GCT​TCC​C
PfMaCFET 3′Homo Rev	TAA​GTC​GAC​GTA​TTA​TTT​CAC​TTG​ACG​TTT​TTT​TAG​ACC
PfMaCFET Guide 1 For	TAT​TAA​TGT​TGA​CTG​CAA​TAA​AGA
PfMaCFETGuide 1 Rev	AAA​CTC​TTT​ATT​GCA​GTC​AAC​ATT
PfMaCFETGuide 2 For	TAT​TAA​AGA​CAC​ATC​AAA​GCG​TTA
PfMaCFETGuide 2 Rev	AAA​CTA​ACG​CTT​TGA​TGT​GTC​TTT
PfMaCFET Geno5 For	CTA​ATT​TTT​TCT​TTG​GAA​TAA​ATT​TTT​ATG​C
PfMaCFET Geno5 Rev	CAT​TTG​TAA​TGT​ATG​CCA​TTT​CAT​CG
PfMaCFET Geno3 For	AAT​TGG​ACG​CAA​TCG​AGC​AGG
PfMaCFET Geno3 Rev	ACA​CAA​AAA​AGT​GAA​ATG​CTA​AAT​ATT​ATA​CT
**Oligonucleotides used for generation of PfMaCFET-GFP parasites**	
PfMaCFET 5GBlock GFP For 2	ATA​CTA​GTA​TAG​CTA​GCT​ATG​GAA​AGC​ATG​TAA​ATA​TTG​ATA​ATA​TTA​TGA
PfMaCFET 5GBlock GFP Rev 2	AGT​TCT​TCT​CCT​TTA​CTC​ATT​TTT​TGA​ACA​TCA​TAA​GAT​TTG​ACT​ATC​TTA​ATT​TTT​CTT​ATA​TTC​CAT​AAT​GCT​TTT​ATA​TGA​CGC​TCT​AAA​TCC​TGC​TCG​ATT​GCG​TC
PfMaCFET 3G Block GFP_For	GAA​CTA​TAC​AAA​GGG​TAA​GCG​GCC​GCT​TAA​TTC​AAA​AAA​AAC​ATA​TAT​ATA​TAT​ATA​TAT​GTA​TAT​TAT​AG
PfMaCFET 3G Block GFP Rev	CGG​TCA​TGA​ATT​CCT​CGA​GCG​GCC​GCG​TAT​TAT​TTC​ACT​TGA​CGT​TTT​TTT​AG
PfMaCFET Guide5 For	TAT​TAA​AGA​CAC​ATC​AAA​GCG​TTA
PfMaCFET Guide5 Rev	AAA​CTA​ACG​CTT​TGA​TGT​GTC​TTT
GFP For	ATCGTCGACATGAGTAAAGGAGAAGAACTTTTCACTGG

### Measurement of Asexual Blood Stage Growth and Gametocyte Development

To compare asexual blood stage replication and growth between the WT *Pf*NF54 and *Pfmacfet¯* parasites, synchronized parasites were set up at an initial ring stage parasitemia of 1% and cultured in 6-well plates and thin smears were prepared at 48 and 96 h. Parasite cultures were diluted at 48 h by the same fold to avoid overgrowth and stressing. Preparation of Giemsa-staining and parasitemia was scored per 1,000 erythrocytes. For calculation, the fold dilution was taken into account. For example: if a culture was dilution to 1% from 6% parasitemia at end of 48 h, and the parasitemia at 96 h was 5%, then estimated parasitemia at 96 h would be 30%.

To compare gametocyte formation between WT *Pf*NF54 and *Pfmacfet¯*, gametocytes were cultured as described above; parasites were removed on day 15 of *in vitro* culture for preparation of Giemsa-stained thin blood smears and gametocytemia was scored per 1,000 erythrocytes.

To obtain activated gametocytes and free gametes for IFAs, mature stage V gametocytes were activated by adding O^+^ human serum and dropping the temperature from 37°C to room temperature as described elsewhere (Singh et al., 2020).

### Generation of Genetic Cross Parasites

For performing genetic crosses, WT *Pf*NF54, *Pfmacfet¯* and *Pfcdpk4¯* were cultured separately to stage V gametocytes, mixed in equal ratio of gametocytemia, and fed to female *A. stephensi* mosquitoes as described above. Mosquitoes were dissected day 7 post-feeding and midguts were digested to isolate genomic DNA following manufacturer’s instructions from the QIAamp DNA Blood Kit. Transmission of *Pfmacfet¯* parasites was determined by genotyping PCRs (Figure 6C).

### Indirect Immunofluorescence

For IFAs on gametocytes and exflagellating gametes, thin smears were prepared on Teflon coated slides and fixed with 4% paraformaldehyde/0.0025% glutaraldehyde solution for 30 min. Slides were kept in a humidity chamber for each step. Fixed parasites were washed twice with PBS and permeabilized using 0.1% Triton X-100/PBS solution for 10 min. Parasites were washed twice with PBS for 5 min each and blocked with 3%BSA/PBS for 45 min. Primary antisera in 3% BSA/PBS was added to the parasites and slides were incubated at 4°C. Antigens were visualized using anti-species antibodies. Images were obtained using a 100 ×1.4 NA objective 90 (Olympus) on a Delta Vision Elite High-Resolution Microscope (GE Healthcare Life Sciences).

### Statistical Analysis

All data are expressed as mean ± SD. Statistical differences were determined using one-way ANOVA with *post hoc* Bonferroni multiple comparison test or unpaired two-tailed Student’s t test, as indicated. Values of *p* < 0.05 were considered statistically significant. Significances were calculated using GraphPad Prism 8 and are represented in the Figures as follows: ns, not significant, *p* > 0.05; *p < 0.05; **p < 0.01; ***p < 0.001; ****p < 0.0001.

## Data Availability

The original contributions presented in the study are included in the article/Supplementary Material, further inquiries can be directed to the corresponding authors.
